# Salivary advanced glycated end products, their receptors, and aMMP‐8 in periodontitis patients with varying glycemic levels: A cross‐sectional study

**DOI:** 10.1002/JPER.24-0362

**Published:** 2024-12-04

**Authors:** Julie Toby Thomas, Betsy Joseph, Sajit Varghese, Baiju Kamalasanan Vijayakumari, Timo Sorsa, Matti Mauramo, Sukumaran Anil, Tuomas Waltimo

**Affiliations:** ^1^ Department of Oral and Maxillofacial Diseases University of Helsinki and Helsinki University Hospital Helsinki Finland; ^2^ Department of Periodontics Saveetha Dental College and Hospitals, Saveetha Institute of Medical and Technical Sciences Chennai India; ^3^ Department of General Medicine Pushpagiri Institute of Medical Sciences and Research Centre Thiruvalla Kerala India; ^4^ Department of Statistics Women's College Trivandrum Kerala India; ^5^ Department of Dental Medicine Karolinska Institutet Stockholm Sweden; ^6^ Department of Pathology University of Helsinki, and Helsinki University Hospital Helsinki Finland; ^7^ Oral Health Institute Hamad Medical Corporation Doha Qatar; ^8^ College of Dental Medicine Qatar University Doha Qatar; ^9^ Department of Medicine University of Basel Basel Switzerland

**Keywords:** active‐matrix metalloproteinase‐8, Type 2 diabetes mellitus, enzyme‐linked immunosorbent assay, glycated hemoglobin, advanced glycation end products, periodontitis, receptor for advanced glycation end products

## Abstract

**Background:**

Advanced glycation end products (AGE) and their receptors (RAGE) have been implicated in developing periodontal complications in diabetic patients. This study aimed to identify salivary AGE, RAGE, soluble RAGE (sRAGE), and active‐matrix metalloproteinase‐8 (aMMP‐8) levels at varying glycemic levels in periodontitis patients.

**Methods:**

Ninety‐eight participants were categorized into uncontrolled DM‐PD group (*n* = 27)—periodontitis patients with uncontrolled Type 2 diabetes mellitus (T2DM) (glycated hemoglobin [HbA1c] ≥7%); controlled DM‐PD group (*n* = 33)—periodontitis patients with controlled T2DM (HbA1c 5.7%–6.9%); SH‐PD group (*n* = 18)—systemically healthy periodontitis patients; and SH‐PH group (*n* = 20)—systemically and periodontally healthy individuals. HbA1c along with the periodontal parameters bleeding on probing (BoP), periodontal probing depth (PPD), clinical attachment loss (CAL), number of missing teeth, and periodontal inflamed surface area (PISA) were estimated. Enzyme‐linked immunosorbent assay (ELISA) was used for analyzing salivary AGE, RAGE, sRAGE, and aMMP‐8. Multiple linear regression analysis was conducted to develop predictive models for HbA1c based on relevant predictor variables.

**Results:**

Periodontitis participants with uncontrolled T2DM exhibited significantly higher BoP, PPD, CAL, number of missing teeth, and PISA, along with elevated AGE, RAGE, and aMMP‐8, compared to other groups (*p* < 0.01). A significant positive association was observed between RAGE and HbA1c levels (*p* < 0.01). Among the predictors, BoP (*p* = 0.046) and CAL (*p* < 0.001) demonstrated a significant positive effect on salivary AGE. PPD was positively associated with RAGE (*p* < 0.05), and BoP was negatively associated with salivary sRAGE levels (*p* = 0.038).

**Conclusions:**

Salivary biomarkers like RAGE and aMMP‐8 exert a potential role in monitoring periodontal health and glycemic control in T2DM patients.

**Plain Language Summary:**

Advanced glycation end products (AGE) and their receptors (RAGE) have been implicated in developing periodontal complications in diabetic patients. This study aimed to identify salivary AGE, RAGE, soluble RAGE (sRAGE), and aMMP‐8 levels at varying glycemic levels in periodontitis patients. Ninety‐eight participants were categorized into Group 1 (*n* = 27)—periodontitis patients with uncontrolled Type 2 diabetes mellitus (T2DM); Group 2 (*n* = 33)—periodontitis patients with controlled T2DM; Group 3 (*n* = 18)—systemically healthy periodontitis patients; and Group 4 (*n* = 20)—systemically and periodontally healthy individuals. Enzyme‐linked immunosorbent assay (ELISA) was used for analyzing salivary AGE, RAGE, sRAGE, and aMMP‐8. The study revealed that participants with uncontrolled T2DM and severe periodontitis exhibited significantly higher levels of salivary AGE, RAGE, and aMMP‐8, along with increased periodontal parameters, compared to controlled T2DM and systemically healthy groups. Conversely, salivary sRAGE levels were significantly lower in the uncontrolled T2DM group. The study also found significant associations between salivary RAGE levels and glycated hemoglobin (HbA1c), as well as between aMMP‐8, AGE, and clinical periodontal parameters. The findings of this study highlight the potential clinical utility of salivary biomarkers, particularly RAGE and aMMP‐8, as noninvasive diagnostic and monitoring tools to evaluate glycemic control and periodontal health in individuals with diabetes.

## 1 INTRODUCTION

Periodontitis is a chronic inflammatory condition with a complex etiology triggered by an imbalanced and dysbiotic dental biofilm, leading to the progressive destruction of tooth‐supporting structures. Diabetes is a prevalent metabolic disorder, broadly categorized into Type 1 diabetes mellitus (T1DM), caused by an absolute deficiency in insulin secretion, and Type 2 diabetes mellitus (T2DM), which is characterized by elevated blood glucose levels due to insufficient insulin secretion or increased tissue resistance. An escalating trend of T2DM has been identified affecting 8.8% of the adult population, with a higher predominance among males, influenced by contributing factors like age, economic growth, urbanization, and sedentary lifestyles.^1^ Undiagnosed cases increase the risk of severe complications, including macrovascular and microvascular diseases, and heightened susceptibility to pathogenic infections like periodontal disease.

The bidirectional relationship between periodontitis and diabetes has been extensively studied. Patients whose diabetes is poorly controlled exhibit faster disease progression and a reduced response to periodontal therapy than those with well‐managed diabetes.^2^, ^3^ Elevated glycated hemoglobin (HbA1c) levels inherently impair immune regulatory mechanisms, resulting in increased pocket depth, clinical attachment loss (CAL), alveolar bone resorption, and tooth loss.^4^, ^5^

Advanced glycation end products (AGE), formed by the nonenzymatic reaction between reducing sugars and proteins, lipids, or nucleic acids, contribute to hyperglycemia, oxidative stress, and inflammation. Increased production of destructive inflammatory cytokines in the serum and gingival crevicular fluid (GCF) due to AGE–RAGE (receptors of AGE) interactions exacerbates the severity of chronic periodontitis.^6^ Elevated AGE expression has been reported in the GCF of patients with chronic periodontitis and T2DM compared to systemically healthy individuals with and without periodontal diseases.^7^

Binding of AGE to their receptors activates pro‐inflammatory pathways in cells, including nuclear factor kappa B (NF‐κB), mitogen‐activated protein kinases (MAPK), and Janus kinase/signal transducer and activator of transcription (JAK/STAT). This exacerbates the secretion of pro‐inflammatory cytokines, chemokines, adhesion molecules, and reactive oxygen species (ROS) by macrophages, endothelial cells, and fibroblasts. Chang et al.^8^ reported increased AGE and RAGE expression in the gingival tissue samples of diabetic compared to nondiabetic periodontitis patients, further emphasizing its potential role in periodontal tissue damage.

Soluble RAGE (sRAGE), a truncated form of the full membrane‐bound RAGE, lacks the transmembrane and cytoplasmic portions that govern RAGE and AGE functions. sRAGE forms complexes with AGE in the systemic circulation, impeding their interaction with membrane‐bound RAGE. Detzen et al.^9^ illustrated a correlation between decreased sRAGE levels and diverse inflammatory circumstances, indicating an escalation in RAGE activity and potential tissue damage.

Recent systematic reviews by Chopra et al.^10^ and Thomas et al.^11^ summarize increased AGE deposition and RAGE activation in both normoglycemic and hyperglycemic patients. Despite evidence of their involvement in various systemic diseases, the mechanism behind the hyperglycemia‐related increase in AGE and RAGE influence on periodontal inflammation remains unclear. Quantifying these biomarkers in oral fluids enables us to understand the role of AGE‐RAGE ligand‐mediated inflammation in diabetic patients with periodontitis.

It is evident that active‐matrix metalloproteinase‐8 (aMMP‐8), but not total MMP‐8 (tMMP‐8), may play a role in differentiating between periodontitis and periodontal health in individuals.^12^ Thus aMMP‐8 is not synonymous to tMMP‐8 in periodontal diagnostics.^13^ aMMP‐8 has been identified to be a feasible biomarker for screening prediabetic/diabetic conditions as an adjunct to a validated questionnaire or combination of risk factors like body mass index (BMI), increasing age, and stage of periodontitis.^14^, ^15^ The present investigation aims to determine the significance of the alternative hypothesis suggesting that the salivary levels of AGE, RAGE, sRAGE, and aMMP‐8 vary with glycemic levels in periodontitis patients with and without T2DM. Furthermore, the study evaluates the correlation between salivary biomarkers, clinical periodontal parameters, and HbA1c levels.

## 2 MATERIALS AND METHODS

### 2.1 Study design and participants

Ninety‐eight participants aged 25–55 years were randomly recruited from the outpatient periodontology department at Pushpagiri Institute of Medical Sciences and Research Centre, Kerala, India, between May 2023 and December 2023 for this cross‐sectional study. The sample size (*n*) was calculated based on a previous study^16^ using the formula *n* = (*Zp*(1 − *p*)/*e*)^2^, where the standard normal ordinate *Z* is 1.96, corresponding to a 95% confidence level, the proportion of T2DM patients with poor glycemic control having severe periodontitis *p* is 61%, and the margin of error *e* is 10%. The study design was approved by the Ethics Committee of Pushpagiri Institute of Medical Sciences and Research Center (institutional review board [IRB] study reference no. 20/0112023). It was executed as per the ethical principles of medical research outlined in the revised version of the Declaration of Helsinki 2013.

The study included periodontitis patients with a history of T2DM, systemically healthy individuals with periodontitis, and systemically and periodontally healthy subjects who had never smoked. Further inclusion criteria were the presence of 20 teeth or more and consent to participate. For the study, the age group 25–55 years was targeted, which has a higher risk for both conditions, taking into account age‐related confounding factors. Exclusion criteria included any history of systemic disorders such as T1DM, hypertension, or cardiovascular disease; liver or kidney disorders; malignancies; bone disorders; autoimmune and oral mucosal diseases; pregnancy; and use of MMP‐8 inhibitors such as tetracycline, chlorhexidine, and bisphosphonates,^17^ nonsteroidal anti‐inflammatory drugs, and antibiotics in the past 6 months. Additionally, individuals with periodontal therapy within the last 6 months, those with removable or fixed partial dentures or orthodontic devices, current or former smokers, tobacco chewers, and those with a BMI exceeding 30 were also not eligible for inclusion in this investigation.

Patients who disagreed with the study protocol, including those who did not consent to blood tests (HbA1c and fasting blood sugar [FBS] levels) or medical consultation, were excluded from the study.

### 2.2 Diagnostic criteria for patient enrollment

The diagnostic criteria for periodontitis (stage 3 or 4) included interdental, buccal, or oral CAL ≥ 5 mm, periodontal probing depth (PPD) ≥6 mm, and radiographic bone loss up to the middle third of the root in more than 30% of sites.^18^ Clinical assessment and evaluation of alveolar bone loss using digital panoramas was performed by a single calibrated periodontist (J.T.T.). Nonperiodontal causes of gingival recession, presence of distomolar pockets as a result of third molar extraction, endodontic lesions, vertical root fractures, and dental caries were excluded from the cases of periodontitis. Participants were considered periodontally healthy if PPD was ≤3 mm, bleeding on probing (BoP) was <10%, and there was no radiographic bone loss.^19^

Participants were advised to undergo routine medical consultations by a physician (S.J.) to identify the presence of T2DM. HbA1c results taken within the past 2 months were considered for categorizing patients into controlled (HbA1c 5.7%–6.9%, 39–52 mmol/mol) or uncontrolled T2DM (HbA1c > 7%, 53 mmol/mol). A single HbA1c measurement within this period, along with FBS at the time of the study, was used to determine glycemic control status. Participants who did not report any previous history of diabetes and had an HbA1c < 5.7% were classified as systemically healthy.

**FIGURE 1:**
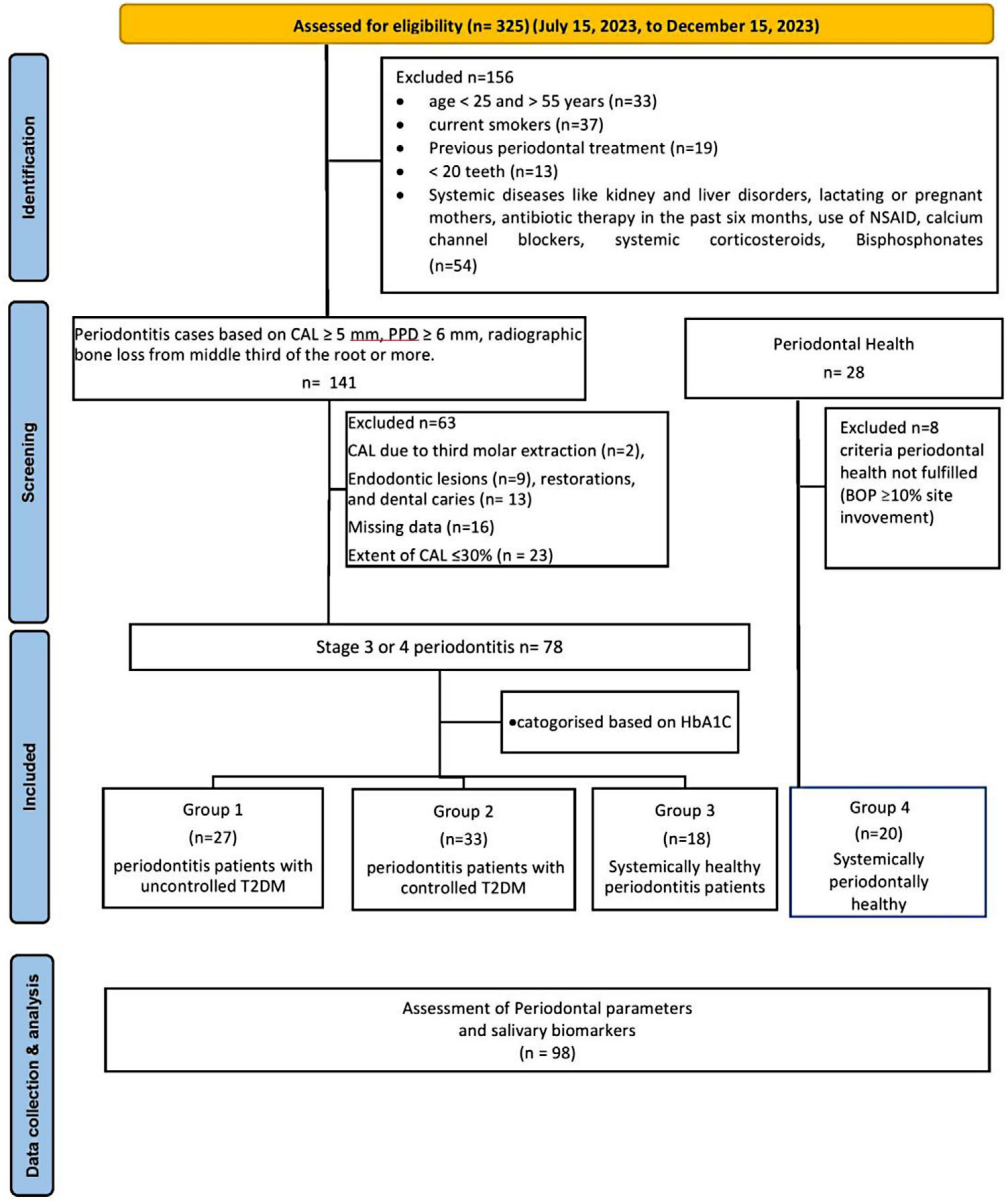
STROBE (Strengthening the Reporting of Observational Studies in Epidemiology) figure on patient recruitment. BoP, bleeding on probing; CAL, clinical attachment loss; HbA1c, glycated hemoglobin; PPD, periodontal probing depth; T2DM, Type 2 diabetes mellitus.

The participants were categorized based on their periodontal and glycemic status as follows:

1. Group 1: Periodontitis patients with uncontrolled T2DM (uncontrolled DM‐PD)
2. Group 2: Periodontitis patients with controlled T2DM (controlled DM‐PD)
3. Group 3: Systemically healthy periodontitis participants (SH‐PD)
4. Group 4: Periodontally and systemically healthy participants or control group (SH‐PH) (Figure 1).

### 2.3 Collection of sociodemographic, anthropometric, and serum parameters

The participant's demographic details, including age, residence, previous medical and dental history, duration of diabetes, tobacco use, medications taken, and routine oral hygiene practices, were collected by a closed‐ended questionnaire (see Supporting Information File 1 “Sociodemographic and oral hygiene questionnaire” in online *Journal of Periodontology*). An investigator (S.J.) conducted the physical examination of all participants and interviewed all participants to verify their responses regarding tobacco use, ensuring that only never smokers and non‐tobacco users were included in the study.

### 2.4 Periodontal clinical examination

The periodontal clinical examination by two calibrated periodontists (J.T.T. and B.J.) included assessment of PPD, CAL, the percentage of sites with BoP, simplified oral hygiene index (S‐OHI), and the number of teeth lost due to periodontitis. The measurements for PPD, CAL, and BoP were assessed at six sites per tooth, except the third molars, using a manual periodontal probe (William's periodontal probe; Hu‐Friedy, Chicago, Illinois, USA).^20^ The S‐OHI was estimated to assess oral hygiene status.^21^ The average PPD and CAL were recorded for every participant. The periodontal inflamed surface area (PISA) was also calculated to quantify the inflammatory burden of periodontitis.^22^ Interexaminer calibration for periodontal parameters was performed in five patients with a manual periodontal probe (William's periodontal probe) in two separate sessions 1 week apart. The recordings were analyzed using the κ‐Cohen test, and a minimum score of 0.80 was considered acceptable.

### 2.5 Saliva collection and processing

Saliva sampling was performed a day after the periodontal examination. Five milliliters of unstimulated whole saliva were obtained from the participants between 8:00 and 10:00 a.m. The participants were advised to pool saliva on the floor of the mouth and to bend their heads slightly downward while sitting erect on the dental chairs with their mouths open into a funnel attached to the sterile collection equipment.^23^ The saliva was drained passively into a sterile graduated container and then stored at −20°C for further analysis.

The sandwich enzyme‐linked immunosorbent assay (ELISA) technique was utilized to estimate the concentration of the biomarkers in the samples according to the manufacturer's guidelines. The stored saliva was thawed and centrifuged (Kenley, London, UK; rotor radius 7 cm) at 3500 RPM for 10 min to remove proteins; the clear supernatant was collected to quantify the biomarkers. Commercially available ELISA kits were used to determine the levels of human active MMP‐8 (catalog no. EK0464; Boster Bio, California, USA), human AGE (catalog no. KBH7931), human RAGE (catalog no. KBH0031), and human sRAGE (catalog no. KBH0027) (KRISHGEN Biosystems, Mumbai, India) according to the manufacturer's instructions. The limit of quantification of aMMP‐8, human AGE, human RAGE, and sRAGE was found to be 30 pg/mL, 38.2 ng/mL, 0.062 ng/mL, and 73 pg/mL (see Supporting Information File 2 “ELISA procedure” in online *Journal of Periodontology*).

### 2.6 Statistical analysis

All data analyses were performed using the statistical package IBM SPSS for Windows, Version 25.0 (released 2017; IBM Corp., Armonk, New York, USA), and the statistical significance was set at a 5% level. The Shapiro–Wilk normality test was used to check the normality of the data. The study summarized continuous variables using mean and standard deviation, while categorical variables were summarized using frequency and percentage. For salivary parameters (aMMP‐8, AGE, RAGE, and sRAGE), one‐way analysis of variance (ANOVA) was used to compare differences among groups, followed by Scheffe's post hoc test for pairwise comparisons.^24^ A *p* value of <0.05 was considered statistically significant. The chi‐square test was used to analyze the differences in categorical characteristics. Pearson's correlation analyses were performed to estimate the correlation between HbA1c and the clinical parameters.

A multiple linear regression analysis was conducted by the statistician (B.K.V.) to develop predictive models for HbA1c based on relevant predictor variables. The adequacy of the regression model was evaluated using adjusted coefficient of determination (*R* ^2^) and *F* values. The analysis included checks for regression assumptions, such as assessing residuals for independence using the Durbin–Watson d statistic, which yielded values of 1.999 and 1.998. Additionally, the normality of residuals was verified with a P–P plot, while homoscedasticity was assessed using a scatter plot of unstandardized residuals. To confirm the absence of multicollinearity, variance inflation factor values were examined, which were found to be below 10. Regression parameter estimates with 95% lower confidence intervals (LCL) and upper confidence intervals (UCL) were also reported.

## 3 RESULTS

### 3.1 Study population

The study participants consisted of 27 uncontrolled DM‐PD patients, 33 controlled DM‐PD patients, 18 SH‐PD patients, and 20 control individuals. No significant difference was observed in age among the groups (*p* > 0.05). Twenty‐one participants in the uncontrolled T2DM group and 22 in the controlled T2DM group were under medication with oral antidiabetic drugs. The distribution of participants among the groups based on demographic characteristics, such as sex and place of stay, was homogenous (*p* > 0.05) (see Table S1 “Sociodemographic characteristics of subjects in study groups” in online *Journal of Periodontology*).

### 3.2 Systemic and periodontal clinical parameters

Table 1 reveals no significant height, weight, or BMI differences among the four groups (*p* > 0.05). ANOVA revealed statistically significant differences in FBS, HbA1c, BoP, S‐OHI, PPD, CAL, number of missing teeth, and PISA scores among all groups (*p* < 0.001). The post hoc test results demonstrate significant pair‐wise differences in all the four groups' clinical periodontal parameters: BoP, S‐OHI, PPD, CAL, number of missing teeth, and PISA score (*p* < 0.001).

**TABLE 1:** Systemic and periodontal parameters of the study subjects.

Variables	Uncontrolled DM‐PD (Group 1)	Controlled DM‐PD (Group 2)	SH‐PD (Group 3)	SH‐PH (Group 4)	*p* value
Number of participants (*n*)	27	33	18	20	
Height (m)	1.58 ± 0.03	1.59 ± 0.02	1.61 ± 0.06	1.60 ± 0.03	0.913
Weight (kg)	70.89 ± 2.20	67.91 ± 1.71	64.67 ± 2.29	70.52 ± 2.94	0.247
BMI (kg/m^2^)	29.17 ± 0.79	26.49 ± 0.79	27.74 ± 0.71	27.79 ± 1.27	0.157
FBS (mg/dL)	193.85 ± 7.87^a^	128.67 ± 2.23^b^	101.00 ± 2.59^c^	97.20 ± 5.27^d^	<0.001^*^
HbA1c	8.53 ± 0.25^a^	6.35 ± 0.07^b^	5.07 ± 0.08^c^	4.98 ± 0.09^d^	<0.001^*^
BoP	61.56 ± 5.38^a^	41.58 ± 4.36^b^	51.11 ± 7.35^c^	7.94 ± 0.39^d^	<0.001^*^
S‐OHI	2.10 ± 0.20^a^	1.82 ± 0.15^b^	2.43 ± 0.25^c^	1.18 ± 0.22^d^	0.001^*^
PPD (mm)	6.74 ± 0.40^a^	5.45 ± 0.18^b^	5.89 ± 0.20^c^	3.00 ± 0.13^d^	<0.001^*^
CAL (mm)	8.22 ± 0.52^a^	6.79 ± 0.30^b^	7.67 ± 0.35^c^	0.85 ± 0.32^d^	<0.001^*^
Number of missing teeth (*n*)	2.81 ± 0.51^a^	2.58 ± 0.51^b^	2.44 ± 0.61^c^	0.15 ± 0.15^d^	0.002^*^
PISA score (cm^2^)	8.95 ± 1.11^a^	6.08 ± 0.77^b^	5.87 ± 1.01^c^	0.60 ± 0.79^d^	<0.001^*^

*Note*: One‐way analysis of variance (ANOVA) was used for continuous variables to analyze differences across the three groups, and Scheffe's post hoc test was used for pairwise differences between groups. Values with different superscript letters indicate a statistically significant pairwise difference (*p *< 0.05) by Scheffe's post hoc test. Values with the same superscript letters indicate that there is no statistically significant pairwise difference (*p *> 0.05) between the corresponding pairs of values.

Abbreviations: BMI, body mass index; BoP, bleeding on probing; CAL, clinical attachment loss; DM, diabetes mellitus; FBS, fasting blood sugar; HbA1c, glycated hemoglobin; PD, periodontitis; PH, periodontally healthy; PISA, periodontal inflamed surface area; PPD, periodontal probing depth; SH, systemically healthy; S‐OHI, simplified oral hygiene index.

* Statistically significant at 1% level (*p *< 0.01).

Group 1 participants (uncontrolled DM‐PD) had significantly higher levels in periodontal parameters, except for S‐OHI, which was significantly higher in Group 3 (SH‐PD) (*p* < 0.001). Specifically, uncontrolled DM‐PD had the highest BoP, PPD, CAL, number of missing teeth, and PISA score, suggesting more severe periodontal disease. At the same time, the SH‐PH group (Group 4) had the lowest values, indicating periodontal health.

### 3.3 Biochemical parameters

The difference in the levels of salivary aMMP‐8, AGE, RAGE, and sRAGE was statistically significant among the groups (*p* < 0.001). aMMP‐8, AGE, and RAGE levels were highest in Group 1 (uncontrolled DM‐PD) (28.10 ng/mL ± 0.35, 6190.29 ng/mL ± 74.42, 19.67 ng/mL ± 0.69). These levels progressively decreased in the other groups, with Group 4 (SH‐PH) having the lowest levels, indicating better health. The mean levels of sRAGE in saliva were highest in Group 2 (controlled DM‐PD) (474.85 pg/mL ± 23.62), followed by Group 4 (SH‐PH) and Group 3 (SH‐PD), and the least in Group 1 (uncontrolled DM‐PD) (*p* < 0.001) (Table 2).

**TABLE 2:** Salivary parameters of the study subjects.

Variables	Uncontrolled DM‐PD (Group 1)	Controlled DM‐PD (Group 2)	SH‐PD (Group 3)	SH‐PH (Group 4)	*p* value
Number of participants (*n*)	27	33	18	20	NA
aMMP‐8 (ng/mL)	28.10 ± 0.35^a^	25.56 ± 0.47^b^	26.54 ± 0.45^c^	12.98 ± 0.31^d^	<0.001^*^
AGE (ng/mL)	6190.29 ± 74.42^a^	5692.54 ± 103.43^b^	5907.97 ± 119.03^c^	2980.40 ± 152.92^d^	<0.001^*^
RAGE (ng/mL)	19.67 ± 0.69^a^	11.33 ± 0.78^b^	9.71 ± 0.45^c^	6.97 ± 0.24^d^	<0.001^*^
sRAGE (pg/mL)	474.85 ± 23.62^a^	658.12 ± 19.42^b^	583.85 ± 33.46^c^	615.85 ± 15.15^d^	<0.001^*^

*Note*: One‐way analysis of variance (ANOVA) was used for continuous variables to analyze differences across the three groups, and Scheffe's post hoc test was used for pairwise differences between groups. Values with different superscript letters indicate a statistically significant pairwise difference (*p *< 0.05) by Scheffe's post hoc test. Values with the same superscript letters indicate that there is no statistically significant pairwise difference (*p* > 0.05) between the corresponding pairs of values.

Abbreviations: AGE, advanced glycation end products; aMMP‐8, active‐matrix metalloproteinase‐8; DM, diabetes mellitus; NA, not applicable; PD, periodontitis; PH, periodontally healthy; RAGE, receptors of AGE; SH, systemically healthy; sRAGE, soluble receptors of AGE.

* Significant at 1% (*p* < 0.01).

### 3.4 Correlations between levels of HbA1c and periodontal parameters

**FIGURE 2:**
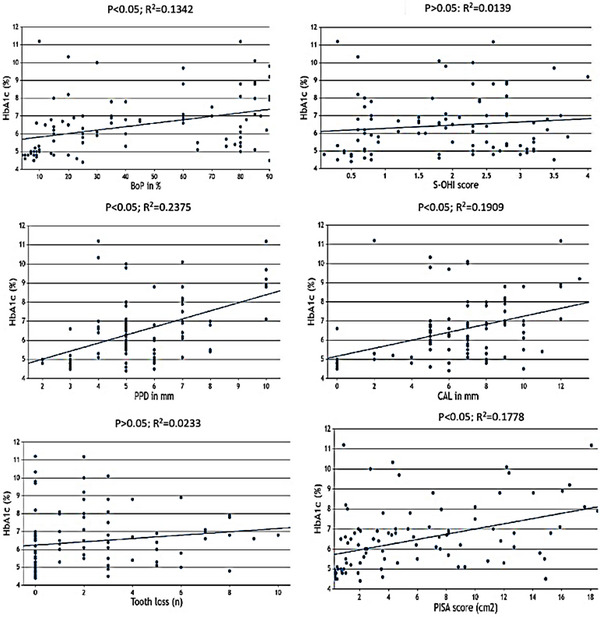
Scatter plots showing correlations. BoP, bleeding on probing; CAL, clinical attachment loss; HbA1c, glycated hemoglobin; PISA, periodontal inflamed surface area; PPD, periodontal probing depth; S‐OHI, simplified oral hygiene index.

The estimated Pearson's correlation coefficient observed between the levels of HbA1c (%) and BoP, PPD, CAL, and PISA scores was 0.13, 0.24, 0.19, and 0.18, respectively (*p* < 0.05). In contrast, the association between S‐OHI and the number of teeth lost with HbA1c levels was not found to be statistically significant (*p* > 0.05) (Figure 2).

### 3.5 Multiple linear regression model analysis between biochemical and HbA1c levels

The multiple linear regression model analysis yielded a reasonable model fit between HbA1c levels and salivary parameters (adjusted *R* ^2^ = 0.509, *F* = 26.2, *p* < 0.01). A significant positive association was found between salivary RAGE levels and HbA1c levels (Table 3).

**TABLE 3:** Multiple linear regression analysis of salivary biochemical parameters on HbA1c levels.

Predictors	Estimates	95% LCL	95% UCL	*p* value
Intercept	3.736	2.1275	5.3436	<0 .001^*^
aMMP‐8 (ng/mL)	2.70e‐4	−6.30e‐4	0.0012	0.552
AGE (ng/mL)	1.02e‐4	−2.97e‐4	5.01e‐4	0.613
RAGE (ng/mL)	0.161	0.1096	0.2116	< 0.001^*^
sRAGE (pg/mL)	−8.26e‐4	−0.0027	0.0010	0.376

*Note*: *n* = 98; adjusted *R* ^2^ = 0.509, *F* = 26.2.

Abbreviations: AGE, advanced glycation end products; aMMP‐8, active‐matrix metalloproteinase‐8; HbA1c, glycated hemoglobin; LCL, lower confidence interval; RAGE, receptors of AGE; sRAGE, soluble receptors of AGE; UCL, upper confidence interval.

* Significant at 1% (*p *< 0.01).

### 3.6 Multiple linear regression model analysis between biochemical and periodontal parameters

The regression analysis yielded a reasonable model fit (adjusted *R* ^2^ = 0.600, *F* = 25.2, *p* < 0.01) between salivary aMMP‐8 and periodontal parameters. Among the predictors, BoP (*p* < 0.05) and CAL were found to have a significant positive association with aMMP‐8 (*p* < 0.01). In contrast, a significant negative association was found between PISA scores (*p* < 0.05) and aMMP‐8 levels (Table 4).

**TABLE 4:** Multiple regression analysis of clinical periodontal variables on salivary parameters.

Predictors	Salivary biomarkers	Estimates	LCL	UCL	*p* value
Intercept	aMMP‐8 (ng/mL)	1546.069	1257.775	1834.36	< 0.001^*^
BoP (%)		5.547	−0.262	11.36	0.048^**^
S‐OHI (1–3)		−75.746	−174.439	22.95	0.131
PPD (mm)		7.444	−81.093	95.98	0.868
CAL (mm)		152.507	99.983	205.03	< 0.001^*^
Number of missing teeth		0.638	−34.156	35.43	0.971
PISA score (cm^2^)		−39.493	−71.015	−7.97	0.015^**^
Intercept	AGE (ng/mL)	3561.4	2911.657	4211.2	< 0.001^*^
BoP (%)		13.3	0.242	26.4	0.046^**^
S‐OHI (1–3)		−109.3	−331.730	113.2	0.332
PPD (mm)		−31.2	−230.755	168.4	0.757
CAL (mm)		325.5	207.103	443.9	< 0.001^*^
Number of missing teeth		34.5	−43.942	112.9	0.385
PISA score (cm^2^)		−87.7	−158.765	−16.7	0.016^**^
Intercept	RAGE (ng/mL)	5.2974	1.4238	9.171	0.008
BoP (%)		0.0537	−0.0243	0.132	0.175
S‐OHI (1–3)		−0.6872	−2.0133	0.639	0.306
PPD (mm)		1.1146	−0.0750	2.304	0.049^**^
CAL (mm)		0.0952	−0.6105	0.801	0.789
Number of missing teeth		0.1258	−0.3417	0.593	0.594
PISA score (cm^2^)		−0.1185	−0.5420	0.305	0.580
Intercept	sRAGE (pg/mL)	668.104	572.69	763.514	< 0.001^*^
BoP (%)		−2.033	−3.96	−0.110	0.038^**^
S‐OHI (1–3)		28.910	−3.75	61.572	0.041^**^
PPD (mm)		−23.004	−52.30	6.297	0.122
CAL (mm)		11.064	−6.32	28.447	0.209
Number of missing teeth		1.343	−10.17	12.858	0.817
PISA SCORE (cm^2^)		0.255	−10.18	10.687	0.961

*Note*: *n* = 98.

Abbreviations: AGE, advanced glycation end products; aMMP‐8, active‐matrix metalloproteinase‐8; BoP, bleeding on probing; CAL, clinical attachment loss; LCL, lower confidence interval; PISA, periodontal inflamed surface area; PPD, periodontal probing depth; RAGE, receptors of AGE; S‐OHI, simplified oral hygiene index; sRAGE, soluble receptors of AGE; UCL, upper confidence interval.

* Significant at 1% (*p *< 0.01).

** Significant at 5% (*p *< 0.05).

The regression analysis yielded a reasonable model fit (adjusted *R* ^2^ = 0.604, *F* = 23.2, *p* < 0.01) between salivary AGE and clinical periodontal variables. Among the predictors, BoP (*p* = 0.046) and CAL (*p* < 0.001) demonstrated a significant positive association with salivary AGE levels, while the PISA score (*p* = 0.016) showed a negative association (Table 4). The regression analysis yielded a reasonable model fit (adjusted *R* ^2^ = 0.218, *F* = 5.50, *p* < 0.01) between salivary RAGE and clinical periodontal parameters. PPD had a significant (*p* < 0.05) positive association with RAGE (Table 4). The regression analysis yielded a reasonable model fit (adjusted *R* ^2^ = 0.118, *F* = 3.15, *p* < 0.01) between sRAGE and clinical periodontal parameters. Among the predictors, BoP (*p* = 0.038) and S‐OHI (*p* = 0.041) showed significant effects on salivary sRAGE levels (Table 4).

## 4 DISCUSSION

The pathobiological concept behind the perturbation of periodontal tissue destruction among the T2DM population has been explored intensively in the literature. Apart from diabetes, the production of AGE has also been reported during aging, smoking, chronic inflammatory conditions, and systemic disorders such as cardiovascular and neurological diseases and cancer.^25^, ^26^ The present cross‐sectional study among the Indian population intended to explore the expression of salivary AGE and its receptors among periodontitis participants diagnosed with varying glycemic levels and to identify these biomarkers' association with periodontal parameters and HbA1c levels.

### 4.1 Periodontal parameters among varying glycemic levels

Participants in Group 1 with uncontrolled DM‐PD consistently exhibited the highest BoP, PPD, CAL, and PISA scores compared to other groups. This underpins the detrimental impact of poorly controlled diabetes on periodontal health, leading to increased inflammation, PPD, CAL, and overall disease severity. A positive association between HbA1c levels and periodontal parameters infers the relevance of glycemic levels on periodontal tissue destruction. This study's findings corroborate those of Rahim et al.^4^ who reported an increase in periodontal parameters in uncontrolled T2DM patients compared to nondiabetic participants and controlled T2DM. It is interesting to highlight that those participants in the controlled DM‐PD group demonstrated better oral hygiene and similar or even better periodontal parameters compared to the SH‐PD group. These findings suggest that with appropriate glycemic control along with oral care individuals with diabetes can maintain periodontal health comparable to those without systemic conditions.^27^ This observation underscores the critical role of hyperglycemia in periodontal health and emphasizes the potential benefits of reasonable glycemic control in managing periodontal disease in diabetic patients. It also suggests that well‐controlled diabetes may not necessarily lead to worse periodontal outcomes than systemic health, provided good oral hygiene practices are maintained.

### 4.2 Salivary aMMP‐8, AGE, RAGE, and sRAGE levels among varying glycemic levels

Saliva, a biological fluid, has been explored as “a window on health status,” which could be a potential diagnostic tool to distinguish systemically healthy subjects from those with pathological conditions. This study revealed statistically significantly elevated aMMP‐8 levels in Group 1 periodontitis participants with uncontrolled T2DM, along with increased periodontal parameters, compared to other groups. Grigoriadis et al.^14^, ^15^ and Miller et al.^28^ reported that mouth rinse and salivary aMMP‐8 levels in T2DM patients can differentiate between post‐treatment periodontal infection resolution and disease severity, making it a reliable test for comparing the condition to nondiabetic patients.

Elevated AGE levels in uncontrolled T2DM, controlled T2DM with periodontal disease, and systemically healthy severe periodontitis, when compared to the control group, demonstrate that hyperglycemia exacerbates inflammation and tissue damage. Substantiative studies by Yoon et al.^29^ and Akram et al.^7^ reported higher AGE concentrations in saliva and GCF of diabetic patients with periodontitis and systemically healthy periodontitis individuals. Experimentally induced periodontitis demonstrated significant changes in serum AGE levels, identifying its role in the pathogenesis of periodontitis.^30^

RAGE belongs to the immunoglobulin superfamily of cell surface receptors that bind to a broad range of ligands, and AGE is one of them. This study confirms strong RAGE expression in the gingival tissue of diabetic patients with periodontitis and systemically healthy patients with severe periodontitis, indicating activation without hyperglycemia.^31^, ^32^ The study revealed that systemically healthy periodontitis patients had higher AGE and RAGE salivary levels, consistent with previous research on their expression in inflamed periodontal tissues. An elevated expression of RAGE contributes to concerns regarding the influence of periodontal inflammation on matrix glycation.^33^

Uncontrolled T2DM patients followed by SH‐PD patients demonstrated significantly the lowest sRAGE levels in this study. The current study's findings are consistent with previous reports where decreased expression of sRAGE was reported in periodontitis patients.^9^, ^34^ Decreased sRAGE levels can disrupt the ligand–RAGE axis, exacerbate inflammation, and cause tissue damage, suggesting sRAGE's potential role in identifying periodontitis severity, unlike previous reports of higher levels among systemically healthy groups.^34^ Elevated sRAGE levels in controlled diabetics may be a compensatory mechanism, acting as a decoy receptor for AGE. This could mitigate the harmful effects of AGE and reflect a decreased inflammatory burden due to local factors. Further investigation is needed to fully understand the complex interplay between glycemic control, periodontal health, and sRAGE levels.

### 4.3 Association between biochemical, glycemic, and periodontal parameters

A significant association of RAGE with HbA1c levels observed in this study implicates the need for further research to elucidate the mechanistic links between salivary RAGE and glycemic control and to explore its clinical utility in monitoring and managing diabetes‐related complications.

Significant positive associations between BoP and CAL with aMMP‐8 have been observed, highlighting the role of aMMP‐8 in periodontal inflammation. This is in agreement with earlier studies which established a better correlation between aMMP‐8 levels in mouth rinse with BoP than plaque index (PI).^35^, ^36^, ^37^ Hence, aMMP‐8 estimation could serve as a valuable tool for monitoring disease progression and therapeutic response in chronic inflammatory conditions. The PISA score demonstrated a positive correlation with HbA1c levels in this study, which is consistent with previous findings.^38^ However, this study observed a negative association between the PISA score and aMMP‐8 and AGE. This could probably be because chronic accumulation of AGE in the extracellular matrix (ECM) can cause vascular stiffness, reducing blood supply to gingival tissues and making them less prone to BoP. AGE can also modulate the expression and activity of MMP and tissue inhibitors of metalloproteinases (TIMP), which play crucial roles in ECM remodeling. Further studies should consider the duration of chronic disease.^39^ Clinical periodontal variables, particularly BoP and CAL, showed significant positive associations with salivary AGE levels, while the PISA score showed a negative estimate (*p* < 0.001). Al‐Sowygh et al.^40^ demonstrated a positive correlation between AGE levels in peri‐implant sulcular fluid (PISF) with PPD and a significant negative correlation with PI among diabetic patients with HbA1c levels >10%.

The current literature reports the lack of an established protocol for AGE estimation, which explains the variation observed in this study compared to previous studies.^41^ Chang et al.^8^ revealed a higher AGE ranking in non‐insulin‐dependent diabetes mellitus (NIDDM) periodontitis patients, with a positive association between AGE ranking and number of missing teeth, but not with PI, PD, or BoP. In the present study, a positive association between AGE and tooth loss was not found to be significant. Studies have demonstrated a significant positive correlation between salivary AGE and BoP in participants with T2DM undergoing orthodontic therapy.^42^, ^43^ The association between PPD and AGE levels in this study was not found to be statistically significant (*p* > 0.05). This indicates that changes in PPD do not predict significant variations in salivary AGE levels. Although PPD reflects periodontal disease severity, its direct influence on salivary levels may be less prominent in this analysis.

The study found a significant positive correlation between PPD and salivary RAGE levels, suggesting that deeper pockets may increase salivary RAGE levels due to increased inflammation in periodontal tissues (*p* = 0.049). The study results contradict previous reports, which found no correlation between RAGE distribution in gingival tissues and PI, PPD, BoP, or number of missing teeth.^8^ However, the association with clinical periodontal variables such as CAL, number of missing teeth, and PISA did not significantly predict changes in salivary RAGE levels (*p* > 0.05). Altingoz et al.^44^ reported that AGE and RAGE estimation in saliva can effectively serve as noninvasive screening markers for periodontitis in diabetic patients.

Higher gingival inflammation, as BoP indicates, is linked to decreased sRAGE levels, while S‐OHI is positively associated with sRAGE levels in saliva. The findings are consistent with a previous study in which a strong negative correlation was found between BoP and sRAGE in serum samples with severe periodontitis with PPD ≥ 5 mm.^45^ The study suggests that sRAGE demonstrates a protective response against inflammation, not influenced by local factors such as PPD, CAL, missing teeth, or PISA score. This is the first study reporting the salivary expression of AGE, RAGE, and sRAGE among controlled and uncontrolled diabetic populations with periodontitis.

A notable strength of this study is its comprehensive approach to selecting T2DM cases based on physician diagnosis, after excluding confounding variables and further categorizing participants into controlled and uncontrolled T2DM groups based on recent HbA1c levels. While this study provides valuable insights into the relationship between salivary biomarkers, glycemic control, and periodontal health, it is essential to consider the generalizability of these findings. The study focuses on the Indian population, which has a high prevalence of T2DM and develops the disease at a younger age and lower BMI compared to Western populations. This could affect AGE levels, receptors, and inflammatory response in periodontal tissues, and dietary habits may influence these factors differently.

This study used a single HbA1c measurement within 3 months and FBS to classify participants' diabetes control status. However, using at least two consecutive HbA1c measurements suggested in clinical guidelines might have provided a more robust assessment of long‐term glycemic control, and future studies might benefit from incorporating multiple measurements. Panoramic X‐rays were utilized for alveolar bone loss assessment, pressing the need for further research using vertical bitewings for improved accuracy.

The current study suggests that oral hygiene practices, dental care access, and awareness of periodontal health may vary significantly among Indian and other populations, potentially impacting the severity and progression of periodontitis. The findings suggest the use of salivary biomarkers for monitoring periodontal health and glycemic control but need validation in diverse populations before global application. Future multicenter studies involving diverse ethnic groups could help establish population‐specific reference ranges for these biomarkers and potentially identify any variations in their diagnostic or prognostic value across different ethnicities.

## 5 CONCLUSION

Our findings indicate that salivary AGE, RAGE, and aMMP‐8 levels are significantly higher in periodontitis patients with uncontrolled glycemic levels. The findings speculate that AGE and their receptors exacerbate the progression of T2DM‐associated periodontitis, whereas the reduced sRAGE expression demonstrated a diminished anti‐inflammatory response. Furthermore, a significant correlation was observed between RAGE and HbA1c levels, as well as between aMMP‐8, and AGE with BoP and CAL. Overall, the study underscores the potential of salivary biomarkers, particularly RAGE and aMMP‐8, in assessing glycemic control and monitoring periodontal health in diabetic individuals with periodontitis. Additionally, future research should focus on validating the clinical utility of salivary AGE, RAGE, and sRAGE as potential biomarkers for early detection, risk assessment, and monitoring the progression of periodontitis in diabetic patients.

## AUTHOR CONTRIBUTIONS

All authors have made substantial contributions to the conception and design of the study. Julie Toby Thomas, Betsy Joseph, and Sajit Varghese collected the data. Baiju K.V. carried out the analysis and interpretated it. Julie Toby Thomas and Sukumaran Anil drafted the manuscript, while Timo Sorsa, Matti Mauramo, and Tuomas Waltimo critically reviewed it. All authors gave final approval for publication.

## CONFLICT OF INTEREST STATEMENT

Author Timo Sorsa is an inventor with US patent 201710023571A1, Japan patent 2016‐554676, and South Korea patent 10‐2016‐7025378. The other authors declare no conflicts of interest.

## Supporting information



File 1: Sociodemographic and Oral Hygiene Questionnaire

File 2: ELISA procedure

Supporting Information

## Data Availability

The data that support the findings of this study are available on request from the corresponding author. The data are not publicly available due to privacy or ethical restrictions.
