# Supplementary material for: Salivary advanced glycated end products, their receptors, and aMMP‐8 in periodontitis patients with varying glycemic levels: A cross‐sectional study
Source: J Periodontol. 2024 Dec 4;96(8):835–47. doi: 10.1002/JPER.24-0362 (PMC12424577; doi:10.1002/JPER.24-0362)
Supplement: Supplementary file 1 — File 1: Sociodemographic and Oral Hygiene Questionnaire [file JPER-96-835-s001.docx]

**SOCIODEMOGRAPHIC AND ORAL HYGIENE QUESTIONNAIRE**

1. Gender (Tick the appropriate answer)

Male Female

2. Age

3. Where is your place of stay? (Tick the appropriate answer)

Rural-urban

4. Height (cm)

5. Weight (kg)

6. Do you suffer from any systemic illness? (Tick the appropriate answer)

No Yes specify

7. Do you take any medication on a regular for the systemic illness?

8. What is your highest educational qualification? (Tick the appropriate answer)

No educational qualifications

Vocational qualification

Degree (e.g., BA, BSc)

Postgraduate qualification

Professional qualification

9. Do you have the habit of smoking? (Tick the appropriate answer)

No Yes occasional

10. Are you employed? No Yes

If yes (Tick the appropriate answer)

1. Employed Full-Time
2. Employed Part-Time
3. Seeking opportunities
4. Retired
5. Prefer not to say

| 1. How many times do you brush your teeth? | No brushing. | Once a day. | | Twice a day or more. |
| --- | --- | --- | --- | --- |
| 2. How do you clean your teeth? | Toothbrush, fluoride toothpaste & dental floss. | Toothbrush, fluoride toothpaste. | | Toothbrush only. |
| 3. How often do you change your toothbrush? | Once in 3 months. | | Once in 6 months or more. | |
| 4. Do you use mouthwashes containing fluoride? | Often. | Sometimes. | | Rare or never. |
| 5. Do you complain of halitosis (bad smell from your mouth)? | Often. | Sometimes. | | Rare or never. |
| 6. Do you complain of  bleeding on brushing or gingival bleeding? | Often. | Sometimes. | | Rare or never. |
| 7. How often do you visit the dental clinic for a check-up? | Once a year or more often. | | Once every few years or when there is pain. | |
| 8. What procedures do you do the most? | Scaling | Restorations | | Extraction |
| 9. How often do you get your teeth cleaned by a dentist? | Rare or never | Once in a year | | Twice in a year |
