# Supplementary material for: Salivary advanced glycated end products, their receptors, and aMMP‐8 in periodontitis patients with varying glycemic levels: A cross‐sectional study
Source: J Periodontol. 2024 Dec 4;96(8):835–47. doi: 10.1002/JPER.24-0362 (PMC12424577; doi:10.1002/JPER.24-0362)
Supplement: Supplementary file 2 — File 2: ELISA procedure [file JPER-96-835-s003.docx]

**ELISA PROCEDURE**

**Biochemical analysis of saliva using Enzyme-linked immune sorbent assay (ELISA)**

*active MMP-8 ELISA Protocol:* 100 𝜇l of samples and standards were used for running the assay. Samples and standards were added onto wells that were precoated with the MMP-8 antibody and incubated at 37 °C for 60 minutes. After 1hr, the sample was aspirated and 100 𝜇l of biotinylated antibody was added and again incubated at 37 °C for 60 minutes. This was then aspirated after 1 hr, washed using wash buffer 2 times, and added with 100 𝜇l of HRP conjugated Streptavidin. This was incubated for 30 minutes at 37 °C. Aspiration and washing were repeated after incubation, added with 100 𝜇l of TMB substrate, and incubated again for 10 minutes at 37 °C. Finally, 100 𝜇L of stop solution was added, and the optical density (OD) was read using an ELISA reader at 450nm immediately, with a detection limit of 30 pg/mL.

*AGE ELISA Protocol:* 50 𝜇l of standards and samples were added into the AGE antibody precoated wells. 50 𝜇l of biotin-antibody was added into these wells and incubated for 60 minutes at 37 °C. Then, the solution was aspirated and washed with wash buffer 2 times. 100 𝜇l of streptavidin-HRP was added into the wells and mixed. The plate was then incubated for 30 minutes at 37 °C. Aspiration and washing were repeated after this incubation. Next, 100 𝜇l of TMB substrate was added and incubated for 10 minutes at 37 °C. Finally, 100 𝜇l of stop solution was added and OD was measured at 450 nm within 10-15 minutes, with the detection limit of 38.2 ng/mL Levels of AGEs were quantified in nanograms per milliliter (ng/mL).

*sRAGE ELISA Protocol:* 50 𝜇l of blank and standards, and 40 𝜇l of samples were added into the wells pre-coated with sRAGE antibody. 10 𝜇l of biotinylated antibody was added into the sample wells. Next, 50 𝜇l of the HRP conjugate was added into all the wells and incubated at 37 °C for 1 hr. After incubation, the wells were aspirated and washed with wash buffer 2 times. Then, 100 𝜇l of TMB substrate was added and incubated at 37°C for 10 minutes. 100 𝜇l of stop solution was added after the incubation to stop the reaction and OD was measured at 450 nm within 10-15 mins, with the detection limit of 73 pg/mL. Levels of sRAGEs were quantified in picograms per milliliter (pg/mL).

*RAGE ELISA Protocol:* 100 𝜇l of samples and standards were added onto wells that were precoated with the RAGE antibody and incubated at 37 °C for 60 minutes. After 1hr, the sample was aspirated and 100 𝜇l of biotinylated antibody was added and again incubated at 37 °C for 60 minutes. This was then aspirated after 1 hr, washed using wash buffer 2 times, and added with 100 𝜇l of HRP conjugated Streptavidin. This was incubated for 30 minutes at 37 °C. Aspiration and washing were repeated after incubation, added with 100 𝜇l of TMB substrate, and incubated again for 10 minutes at 37 °C. The reaction was then stopped by adding 100 𝜇l of stop solution. Absorbance was read at 450 nm on a plate reader and RAGE concentrations were determined from the standard curve, with the detection limit of 0.062 ng/mL. Levels of RAGEs quantified in nanograms per milliliter (ng/mL).
