# Supplementary material for: Salivary advanced glycated end products, their receptors, and aMMP‐8 in periodontitis patients with varying glycemic levels: A cross‐sectional study
Source: J Periodontol. 2024 Dec 4;96(8):835–47. doi: 10.1002/JPER.24-0362 (PMC12424577; doi:10.1002/JPER.24-0362)
Supplement: Supplementary file 3 — Supporting Information [file JPER-96-835-s002.docx]

**Table S1. Sociodemographic characteristics of subjects in study groups**

| **Characteristics** | **Uncontrolled DM-PD (n=27)** | **Controlled DM-PD (n=33)** | **SH-PD (n=18)** | **SH-PH (n=20)** | **p-value** |
| --- | --- | --- | --- | --- | --- |
| **Age in years** (Mean ± SD) | 46.9 ± 1.3 | 48.5 ± 1.1 | 44.8 ± 2.2 | 43.1 ± 8.2 | 0.065 |
| **Gender** |  |  |  |  |  |
| Male | 14 (51.9%) | 18 (54.5%) | 9 (50.0%) | 11 (55.0%) | 0.896 |
| Female | 13 (48.1%) | 15 (45.5%) | 9 (50.0%) | 9 (45.0%) |  |
| **Place of stay** |  |  |  |  |  |
| Rural | 13 (48.1%) | 16 (48.5%) | 9 (50.0%) | 10 (50.0%) | 0.986 |
| Urban | 14 (51.9%) | 17 (51.5%) | 9 (50.0%) | 10 (50.0%) |  |
| **Education** |  |  |  |  |  |
| Primary | 7 (25.9%) | 9 (27.3%) | 5 (27.8%) | 5 (25.0%) | 0.998 |
| Secondary | 11 (40.7%) | 13 (39.4%) | 7 (38.9%) | 8 (40.0%) |  |
| Graduate | 9 (33.3%) | 11 (33.3%) | 6 (33.3%) | 7 (35.0%) |  |
| **Occupation** |  |  |  |  |  |
| Employed | 15 (55.6%) | 18 (54.5%) | 10 (55.6%) | 11 (55.0%) | 0.999 |
| Unemployed | 12 (44.4%) | 15 (45.5%) | 8 (44.4%) | 9 (45.0%) |  |
| **Medication use** |  |  |  |  |  |
| Yes | 21 (77.8%) | 22 (66.7%) | 0 (0%) | 0 (0%) | <0.001** |
| No | 6 (22.2%) | 11 (33.3%) | 18 (100%) | 20 (100%) |  |

**: Significant at 1% (p<0.01); Values are expressed as mean ± standard deviation for continuous variables. Descriptive and categorical variables are expressed as frequency (percentage). One-way analysis of variance (ANOVA) followed by post-hoc Tukey test comparisons was used for continuous variables, and Pearson’s chi-square test was used for categorical variables to analyze significant differences.

DM-PD: Diabetes Mellitus with Periodontitis; SH-PD: Systemically Healthy with Periodontitis; SH-PH: Systemically Healthy and Periodontally Healthy. p-value calculated using the Chi-square test.
